# Predictors of gastrointestinal malignant tumors in fecal occult blood positive individuals prior to percutaneous coronary intervention: a Nomogram-based approach

**DOI:** 10.1515/med-2026-1427

**Published:** 2026-06-01

**Authors:** Yinman Wang, Yaolin Chen, Juying Qian

**Affiliations:** Department of Cardiology, Zhongshan Hospital, Fudan University, Shanghai Institute of Cardiovascular Diseases China, Shanghai, China; State Key Laboratory of Cardiovascular Diseases, Zhongshan Hospital, Fudan University, Shanghai, China; NHC Key Laboratory of Ischemic Heart Diseases, Beijing, China; Key Laboratory of Viral Heart Diseases, Chinese Academy of Medical Sciences, Beijing, China; National Clinical Research Center for Interventional Medicine, Shanghai, China; Department of Cardiology, Zhongshan Hospital, Fudan University (Xiamen Branch), Xiamen, China

**Keywords:** fecal occult blood, percutaneous coronary intervention, endoscopy, gastrointestinal malignant tumor, risk prediction

## Abstract

**Objectives:**

Fecal occult blood (FeOB) is widely used for screening gastrointestinal diseases, but its predictive value for gastrointestinal malignant tumor (GI MT) in patients receiving antiplatelet medication before percutaneous coronary intervention (PCI) remains unclear.

**Methods:**

This retrospective cohort study included 1986 patients with an initial positive FeOB test prior to PCI from January 2015 to April 2020. All patients underwent repeated FeOB testing. If the second test was negative, the patient was classified into the FeOB-negative group. If the second test was positive, a third FeOB test was performed. Patients were then categorized into: 1) FeOB-negative group (negative result on the second or third test), and 2) FeOB-positive group (positive results on all three consecutive tests). According to endoscopic results, the patients were divided into GI MT and non-GI MT groups. Multivariable logistic regression was used to identify independent predictors, and a nomogram was constructed to estimate individualized risk.

**Results:**

Among 1986 patients with an initial FeOB positive, 427 (21.5 %) patients received endoscopy. Endoscopy identified 127 patients with GI MT. The prevalence of GI MT was significantly higher in patients with three consecutive positive FeOB results compared with those whose results converted to negative (39.9 % vs. 14.2 %, p<0.001). Multivariable analysis identified age (OR 1.05, 95 % *CI* 1.02–1.08, p<0.001) and persistent FeOB positivity (OR 3.97, 95 % *CI* 2.32–6.80, p<0.001) as independent predictors. A nomogram incorporating these variables demonstrated acceptable discrimination and clinical utility.

**Conclusions:**

For patients with an initial positive FeOB test before PCI, advanced age and persistent FeOB positivity are associated with increased risk of GI MT. A risk-based approach using repeated FeOB testing and individualized risk estimation may support decisions regarding endoscopic evaluation.

## Introduction

Dual anti-platelet therapy (DAPT) is recommended after percutaneous coronary intervention (PCI) to reduce coronary stent events, such as in-stent restenosis (ISR) and stent thrombosis (ST) [1]. However, gastrointestinal bleeding caused by malignant tumors is the most common reason for DAPT discontinuation after coronary stenting [2]. Furthermore,the necessity for DAPT may delay the timing of digestive tumor surgery because of major bleeding. Therefore, identifying predictors of gastrointestinal malignant tumors (GI MT) is essential for risk stratification before PCI.

The fecal occult blood (FeOB)test using fecal immunochemical test (iFOBT), a feasible and effective screening examination for detecting GI tract diseases, is a routine test before PCI. iFOBT is not influenced by medications, dietary products and any other constituents present in feces [3]. Although iFOBT is noninvasive and low-cost and effective, it has a low positive-predictive value ranging from 6.7 % to 12.8 % [4], with a false negative rate of 11 % for screening colorectal cancer [5]. It is reported that the sensitivity and specificity of one positive FeOB for colorectal cancer were 90.4% and 53.8 %, and those of three positive tests were 53.9 % and 88.5 %, respectively [6]. This suggests that serial testing could mitigate the high false-positive rate. Although endoscopy remains the gold standard for diagnosis, it is invasive, expensive, and suffers from poor patient compliance, with studies reporting that only 42–61 % of individuals with a positive FeOB undergo a complete colon evaluation [7], [8], [9].

Given the high false-positive rate of a single iFOBT and the low compliance for subsequent endoscopy, it is essential to refine the screening process for high-risk GI MT in patients preparing for PCI. We hypothesized that in patients with an initial positive FeOB test before PCI, a strategy of repeat testing combined with clinical risk factors would improve the prediction of GI MT. Therefore, this study aimed to identify independent predictors of GI MT in this specific population and to develop a practical nomogram-based tool to guide the decision for endoscopic examination.

## Materials and methods

### Study population

This retrospective cohort study included individuals with an initial positive FeOB result prior to coronary angiography admitted to Zhongshan Hospital, Fudan University, Shanghai, between January 1, 2015, and April 30, 2020. Participants with a history of digestive malignancy (n=23) were excluded, and 50 patients were lost to follow-up, resulting in 1986 patients (1,544 male, 442 female) in the final cohort.

### Study protocol

All patients with an initial positive FeOB test underwent repeat testing. If the second test was negative, the patient was classified into the FeOB-negative group. If the second test was positive, a third FeOB test was performed. Patients were then categorized into: 1) FeOB-negative group (negative result on the second or third test), and 2) FeOB-positive group (positive results on all three consecutive tests). A comparison of age, gender, and initial FeOB status between the 50 patients lost to follow-up and the 1,986 patients who remained in the study revealed no significant differences (all p>0.05), suggesting the random loss to follow-up was non-differential (Supplementary table S1).

Data collected included age, sex, past history (hypertension, diabetes mellitus, prior PCI, digestive disease history), health-related behaviors (smoking, alcohol consumption), combined medications (proton pump inhibitors, antiplatelets and anticoagulants) and laboratory data (hemoglobin, platelet count, creatinine level).

### FeOB examination

Each FeOB screening consisted of a single fecal sample. Sampling kits were provided with standardized instructions by trained clinicians. Participants didn’t need to follow dietary restrictions or discontinue anticoagulation or antiplatelet treatment ahead of sampling. Samples were delivered to the centralized laboratory at Zhongshan Hospital. All samples were analyzed using fecal occult blood test paper using colloidal gold immunochromatographic assay (Orienter, Sichuan, China). The threshold for a positive FeOB was defined as fecal hemoglobin level >0.2 μg/mL.

### Following-up endoscopy

For patients scheduled for PCI, endoscopic evaluation for a positive FeOB was typically arranged urgently (within 1–2 weeks) to assess the bleeding risk before initiating or continuing antiplatelet therapy. Ultimately, 427 patients underwent endoscopy in total.

### Statistical analysis

Continuous variables were expressed as mean ± standard error (SEM), or as median (interquartile range) according to the distributions examined by the Kolmogorov-Smirnov test. Categorical variables were presented as numbers (proportions). For group comparisons, the two-sided independent samples t-test was used for normally distributed continuous data, and the Mann-Whitney U test was used for non-normally distributed data. The chi-square test or Fisher’s exact test was used for categorical data. The association between variables and GI MT was evaluated by Spearman correlation test. The predictors of GI MT were determined by multivariable logistic regression analyses. The cutoff value of variable for predicting GI MT were determined by the Youden index via receiver operating characteristic (ROC) curve analysis. A multivariable logistic regression model was constructed. The “VRPM” “Rms”, “ggDCA”, “rmda” packages were used to create the nomogram, calibration curve and decision curve (DCA) analysis. Nomogram, calibration curve, and DCA were used to test the clinical applicability of the model. All statistical analyses were two-sided with a given p-value of <0.05 considered statistically significant.

### Ethical statement

The study was conducted in accordance with the Declaration of Helsinki. This study was approved by the ethics committee of Zhongshan Hospital (Approval No.: B2021-726R; Date: 2022-3-3), and informed consent was obtained from all patients for inclusion in the study.

## Results

### Baseline characteristics of the study population

**Figure 1:**
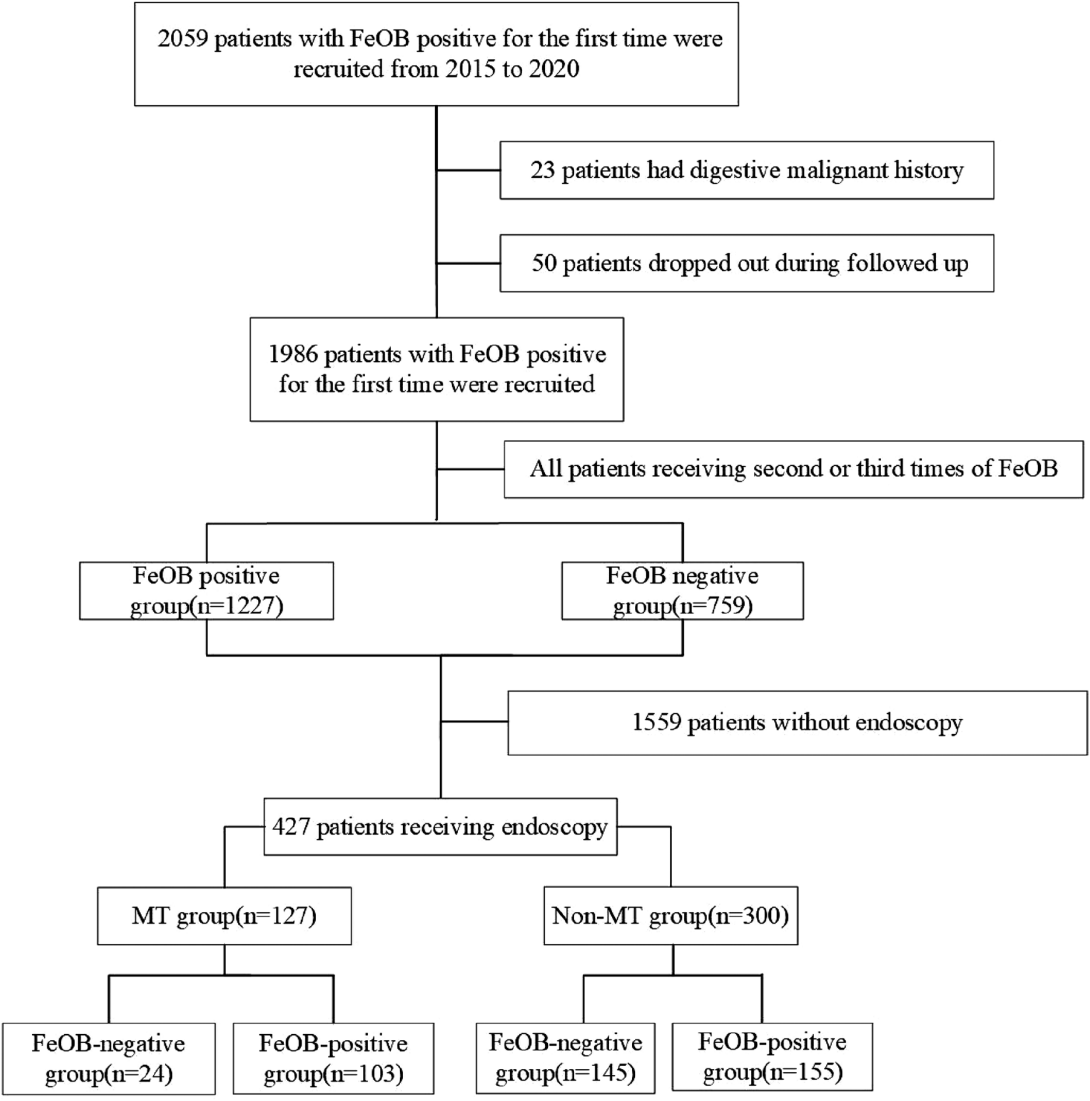
The flow chart of the study population. FeOB, fecal occult blood; MT indicates gastrointestinal malignant tumor.

Of the total 1,986 patients, 1,227 (61.8 %) had three consecutive positive FeOB results. A total of 427 patients had received endoscopy finally (Figure 1). Among them, 169 patients had a negative FeOB result on the second or third test (FeOB-negative group), while 258 had three consecutive positive FeOB results (FeOB-positive group). Baseline characteristics of the two groups are listed in Table 1. There were significant differences between the two groups in usage of proton pump inhibitors, hemoglobin, platelet count and the number of patients who received PCI (p*<*0.05 for all).

**Table 1:** Baseline characteristics of the FeOB-negative and FeOB-positive groups.

	FeOB-positive group (n=258)	FeOB-negative group (n=169)	p-Value
Age, yrs	68.0 (62.0–73.0)	67.0 (60.0–73.0)	*P*=0.14
Male (n, %)	196 (76.0 %)	135 (79.9 %)	*P*=0.41
Hypertension (n, %)	168 (65.1 %)	109 (64.5 %)	*P*=0.92
Diabetes mellitus (n, %)	79 (30.6 %)	47 (27.8 %)	*P*=0.59
Smoking (n, %)	100 (38.8 %)	73 (43.2 %)	*P*=0.37
Alcohol (n, %)	29 (11.2 %)	24 (14.2 %)	*P*=0.37
Digestive disease history (n, %)	54 (20.9 %)	32 (18.9 %)	*P*=0.71
Prior PCI (n, %)	79 (30.6 %)	64 (37.9 %)	*P*=0.14
Atrial fibrillation (n, %)	23 (8.9 %)	10 (5.9 %)	*P*=0.27
Antiplatelet drug (n, %)	174 (67.4 %)	108 (63.9 %)	*P*=0.47
Aspirin	151 (58.8 %)	97 (57.4 %)	*P*=0.84
Cilostazol	8 (3.1 %)	5 (3 %)	*P*=1.00
Clopidogrel	67 (26.0 %)	53 (31.4 %)	*P*=0.23
Ticagrelor	24 (9.3 %)	18 (10.7 %)	*P*=0.74
Oral anticoagulants (n, %)	11 (4.3 %)	5 (3.0 %)	*P*=0.61
Proton pump inhibitors (n, %)	177 (68.6 %)	88 (52.1 %)	^a^ *P<*0.01
Type of angina (n, %)			*P*=0.07
SCAD	194 (75.2 %)	115 (68.0 %)	
UA	38 (14.7 %)	22 (13.0 %)	
NSTEMI	11 (4.3 %)	14 (8.3 %)	
STEMI	15 (5.8 %)	18 (10.7 %)	
Hemoglobin, g/L	121.0 (105.0–138.0)	135.0 (120.0–144.0)	^a^ *P<*0.01
Platelet count (10^9^/L)	210.0 (169.8–263.0)	194.5 (158.0–229.8)	^a^ *P<*0.01
Creatinine, µmol/L	81.0 (71.0–95.0)	82.5 (71.0–96.0)	*P*=0.81
Received PCI (n, %)	57 (22.1 %)	132 (78.1 %)	^a^ *P<*0.01
Gastric disease history (n, %)	54 (20.9 %)	32 (18.9 %)	*P*=0.31

Values were presented in number (percentage) or normally distributed variables as mean ± SEM, non-normally distributed as median (IQR). FeOB, fecal occult blood; SCAD, stable coronary artery disease; UA, unstable angina; NSTEMI, non-ST-elevation myocardial infarction; STEMI, ST-elevation myocardial infarction; PCI, percutaneous coronary intervention;

### Endoscopy

A total of 427 patients received endoscopy (427/1,986, 21.5 %). To avoid selection bias, a comparative analysis results of key baseline characteristics between patients who underwent endoscopy (n=427) and those who did not (n=1,559) were added as a new supplementary table S2. After endoscopy, 103 patients were (39.9 %) diagnosed with GI MT in FeOB-positive group while 24 patients (14.2 %) diagnosed with GI MT in FeOB-negative group. The GI MT rate was significantly different between the two groups (p<0.001). According to endoscopy, patients were divided into GI MT group and non-GI MT group. All characteristics of the two groups were listed in Table 2. There were significant differences between two groups in age, hemoglobin and the number of patients whose FeOB turned negative at second or third times (p*<*0.05 for all).

**Table 2:** Baseline characteristics of the Non-GI MT and GI MT groups.

	Non-GI MT group(n=300)	GI MT group (n=127)	p-Value
Age, yrs	66.5 (60.0–73.0)	69.0 (64.0–75.0)	^a^ *P<*0.01
Male (n, %)	230 (76.7 %)	101 (79.5 %)	*P*=0.61
Hypertension (n, %)	193 (64.3 %)	84 (66.1 %)	*P*=0.74
Diabetes mellitus (n, %)	88 (29.3 %)	38 (29.9 %)	*P*=0.91
Smoking (n, %)	118 (39.3 %)	55 (43.3 %)	*P*=0.45
Alcohol (n, %)	34 (11.3 %)	19 (15.0 %)	*P*=0.34
Prior PCI (n, %)	107 (35.7 %)	36 (28.3 %)	*P*=0.15
Atrial fibrillation (n, %)	23 (7.7 %)	10 (7.9 %)	*P*=1.00
Medication (n, %)			
Aspirin	179 (59.7 %)	69 (54.3 %)	*P*=0.34
Cilostazol	9 (3.0 %)	4 (3.1 %)	*P*=1.00
Clopidogrel	91 (30.3 %)	29 (20.9 %)	*P*=0.13
Ticagrelor	33 (11.0 %)	9 (7.1 %)	*P*=0.29
Oral anticoagulants (n, %)	11 (3.7 %)	5 (3.9 %)	*P*=1.00
Proton pump inhibitors (n, %)	186 (62.0 %)	79 (62.2 %)	*P*=1.00
Hemoglobin, g/L	129.0 (114.0–142.8)	122.0 (104.0–137.0)	^a^ *P<*0.01
Platelet count (10^9^/L)	201.0 (166.0–247.0)	210.0 (169.0–262.0)	*P*=0.14
Creatinine, µmol/L	81 (71.0–96.0)	82.0 (72.0–94.0)	*P*=0.84
Received PCI (n, %)	140 (46.7 %)	49 (38.9 %)	*P*=0.14
FeOB becomes negative at second or third times (n, %)	145 (48.3 %)	24 (18.9 %)	^a^ *P<*0.01

Values were presented in number (percentage) or normally distributed variables as mean ± SEM, non-normally distributed as median (IQR). GI MT, gastrointestinal malignant tumor; PCI, percutaneous coronary intervention; FeOB, fecal occult blood. ^a^p-value<0.05.

**Figure 2:**
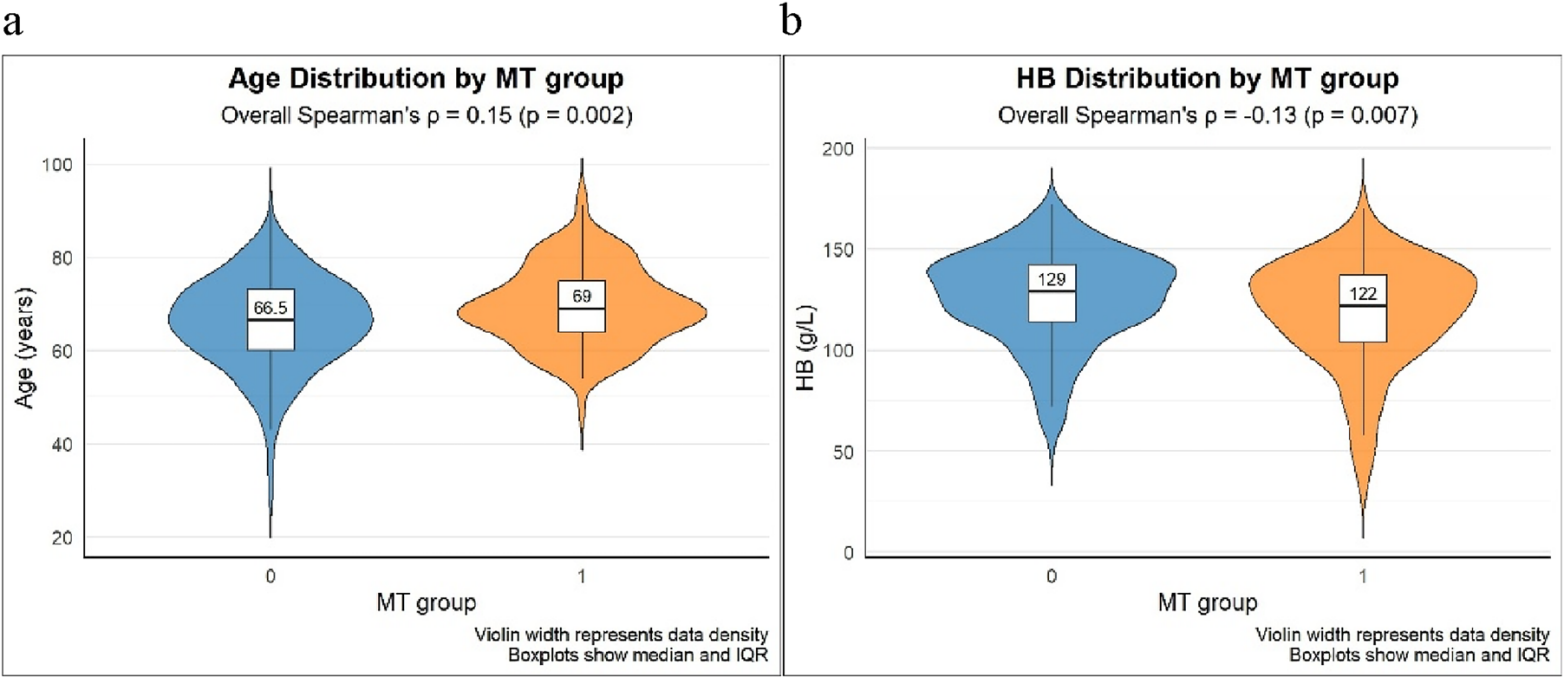
The relationship between age (2a), HB(2b) and GI MT. 1=Gastrointestinal malignant tumor (GI MT); 0=Non-GI MT, MT, gastrointestinal malignant tumor; HB, hemoglobin.

Spearman correlation showed that age was positively associated with GI MT (*r*=0.15, p<0.01) while hemoglobin was negatively associated with GI MT (*r*= −0.13, p<0.01) (Figure 2).

### Predictors for GI MT

**Figure 3:**
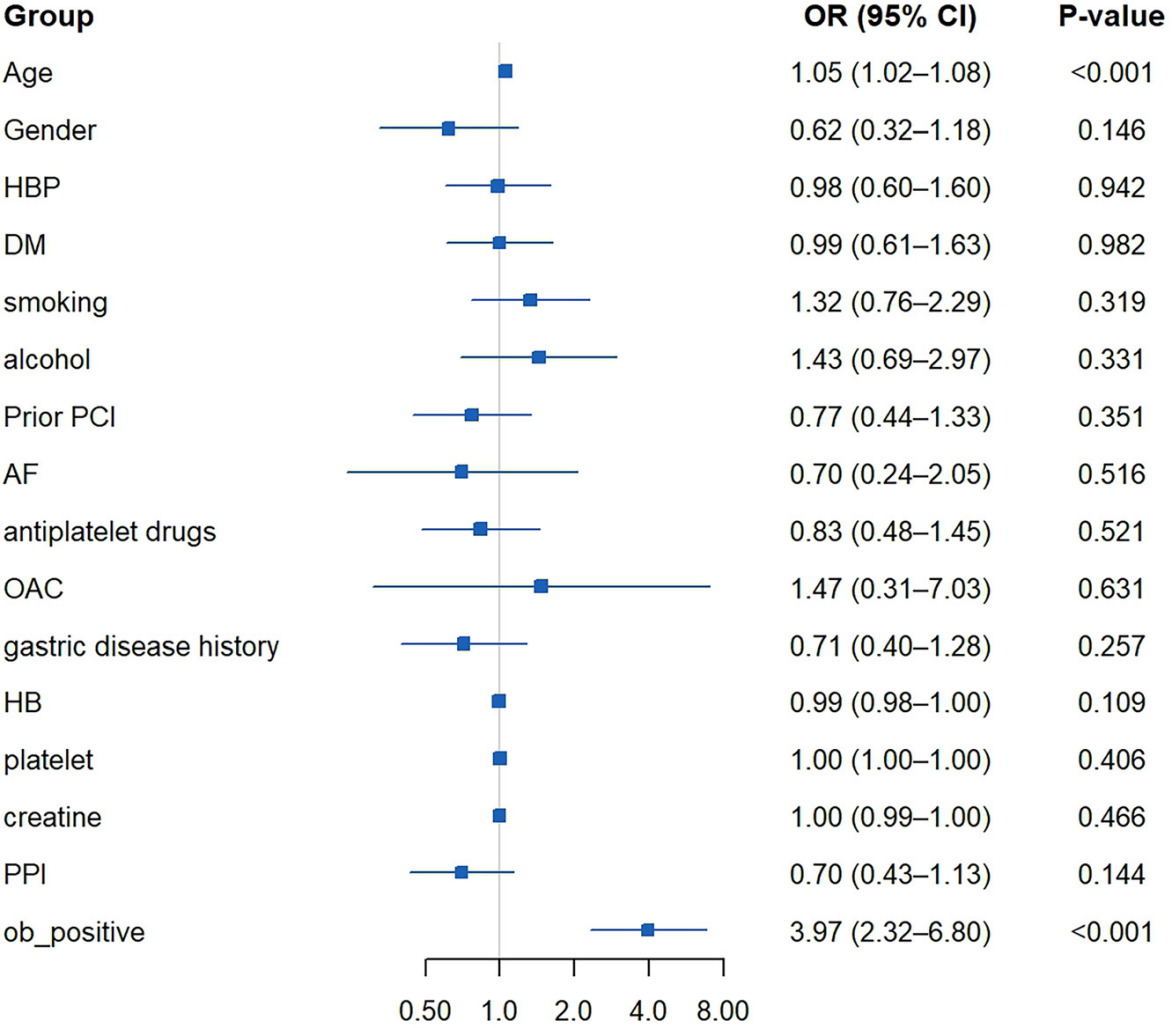
Logistic regressions shows factors associated with GI MT. HBP, hypertension; DM, diabetes mellitus; PCI indicates percutaneous coronary intervention; AF, atrial fibrillation; OAC, oral anticoagulants; HB hemoglobin; PPI, proton pump inhibitors; ob_positive, fecal occult blood positive for three times.

To identify variables with predictive value for GI MT, univariate logistic regression was first applied. A total of 3 independent predictor variables, namely, age, hemoglobin and three consecutive positive FeOB tests during the procedure, were identified (Supplementary table S3). After including confounding factors (Gender, HBP, DM, smoking, alcohol etc.) in this multivariate analysis, age and FeOB test positive were strongly positively correlated with GI MT respectively (OR 1.05, 95 % *CI* 1.02–1.08, p<0.001, OR 3.97, 95 % *CI* 2.32–6.80, p*<*0.001, respectively) (Figure 3).

### Nomogram prediction of GI MT

**Figure 4:**
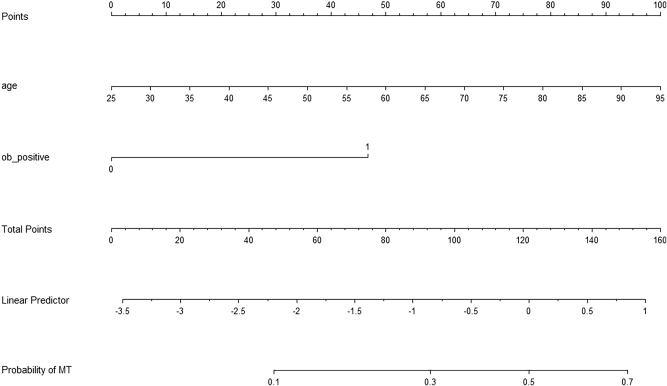
Prediction of GI MT based on the risk nomogram. FeOB positive for three times: 0 no, 1 yes. The patient was 80 years old and FeOB positive for three times. The prediction of GI MT was 52.63 %. GI MT, gastrointestinal malignant tumor; FeOB, fecal occult blood.

Based on the multivariate logistic regression model, a nomogram was developed to predict the probability of GI MT in patients with initial positive FeOB prior to PCI (Figure 4). The nomogram incorporates two predictors: age and FeOB test positivity on all three consecutive tests. For each predictor, a corresponding point value was assigned according to its scale on the nomogram: age (ranging from 25 to 95 years) and FeOB status (coded as 0 for negative on second/third test, and 1 for positive on all three tests). The total points were calculated by summing the individual points from each predictor. These total points were then aligned with the bottom risk scale to obtain the predicted probability of GI MT, ranging from approximately 0.1 to 0.7. The construction approach of this nomogram aligned with methodologies described in similar predictive model studies [10].

**Figure 5:**
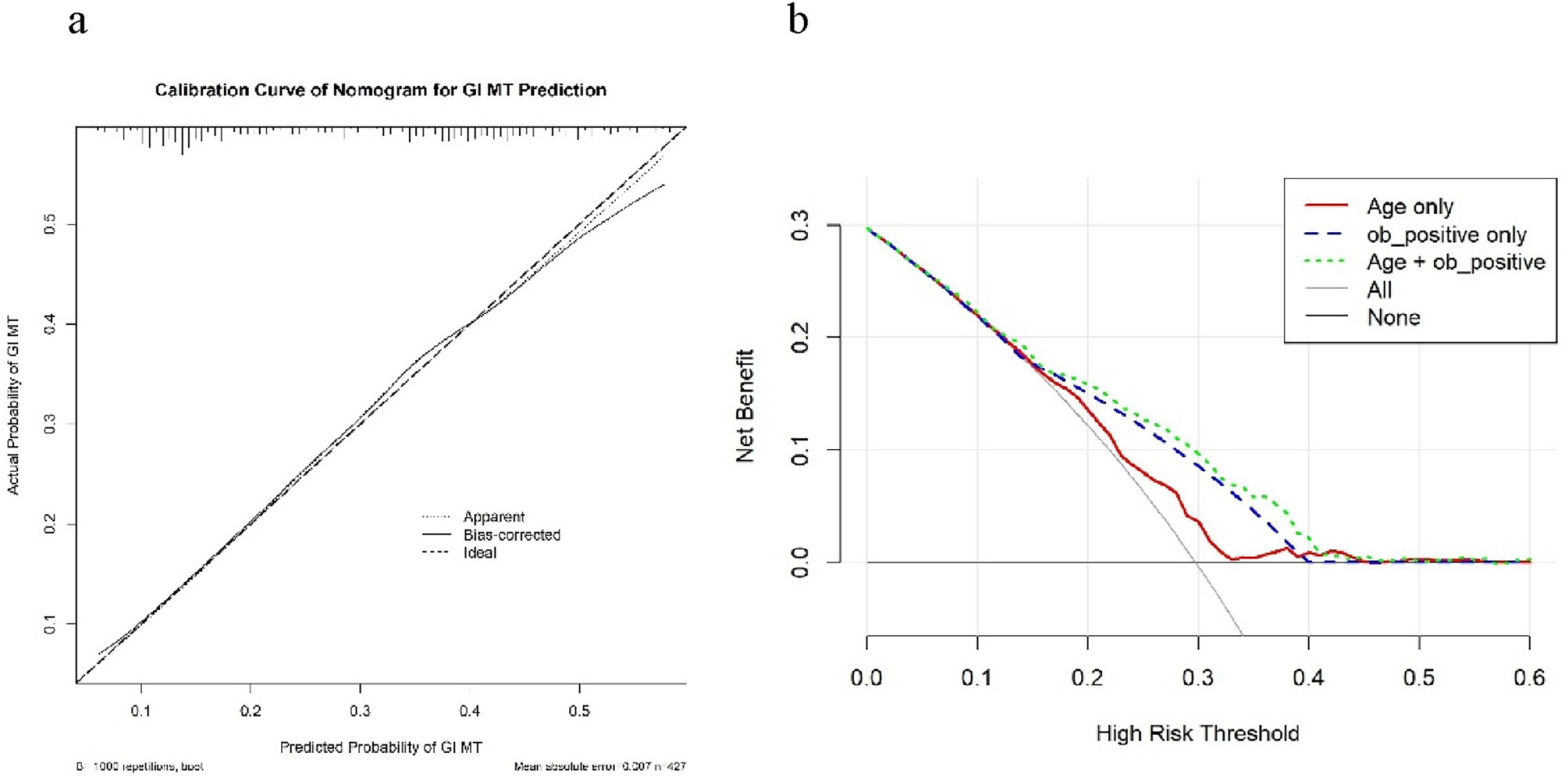
The calibration curve and the decision curve analysis to predict GI MT. The calibration curve (a) showed the predictive efficacy of GI MT in patients before PCI. The decision curve analysis (b) showed the clinical utility of predicting GI MT in patients before PCI based on each individual predictor variable and the combined risk nomogram. GI MT, gastrointestinal malignant tumor; PCI, percutaneous coronary intervention.

The calibration curve for the nomogram predicting GI MT is presented in Figure 5a. The closer the calibration curve (solid line) is to the ideal line (dashed line), the better the agreement between predicted and actual probabilities. In our model, the calibration curve closely approximates the ideal line, indicating that the predicted incidence of GI MT aligns well with the actual incidence. The mean absolute error was 0.007, further confirming good calibration accuracy. A total of 427 samples were included in this analysis, with 1,000 bootstrap repetitions performed to assess stability.

Decision curve analysis (DCA) was conducted to evaluate the clinical net benefit of the nomogram (Figure 5b). The horizontal axis represents the threshold probability, and the vertical axis represents the net benefit. The two reference lines indicate strategies of “intervening in none” (horizontal line) and “intervening in all” (diagonal line). The DCA curve of the nomogram is consistently higher than both reference lines across a wide range of threshold probabilities, indicating that the nomogram provides superior clinical decision support. Notably, net benefit is maintained for threshold probabilities up to 0.4, suggesting the model is clinically useful within this range.

ROC curve analysis demonstrated that age and FeOB had significant diagnostic efficacy for GI MT, with AUC values of 0.65 (95 %*CI* 0.60–0.69) and 0.60 (95 %*CI* 0.54–0.65), respectively. (Figure S1a, Figure S1b). The cutoff of age was 65.5 years on the ROC. When we included both variables in the ROC curve,the AUC was 0.69 (95 %*CI* 0.64–0.75). (Figure S1c). The AUC for the combined model was significantly higher than that for either single variable (p<0.01 for age and p<0.01 for FeOB positive). The AUC for combination of the above two variables, along with PPI usage and hemoglobin levels (Figure S1d) was 0.71 (95 % *CI*: 0.65–0.76). The improvement in AUC was not statistically significant (*P* for comparison=0.15) compared with the AUC for FeOB positive and age.

## Discussion

In the present study, we found that among patients with an initial positive FeOB test before PCI, repeat testing and advanced age were independent predictors of GI MT. Based on these two readily available clinical variables, we developed and validated a nomogram that can effectively estimate an individual patient’s probability of GI MT, providing a practical tool to guide the decision for subsequent endoscopic examination. Our results demonstrated that [1]: the compliance rate for endoscopic examination was not high among FeOB-positive patients [2]. three times FeOB test are essential for the diagnosis of GI MT for patients with FeOB positive before PCI [3]. When the predicted probability of GI MT exceeds 0.2 (20 %), further endoscopic examination is generally recommended.

In our study, among all 1,986 patients, only 427 (21.5 %) underwent endoscopy. This finding is consistent with a cohort study by Grewal et al. [11], which investigated gastrointestinal bleeding in patients undergoing anticoagulation for atrial fibrillation. Among 8,612 patients with gastrointestinal bleeding, only 2,119 (24.6 %) received gastrointestinal endoscopy after the bleeding event. As a result, we need to find a flowchart to select more urgent patients for endoscopy. The low rate of endoscopic examination among FeOB-positive patients indicates a substantial discrepancy between screening detection and diagnostic confirmation in routine clinical practice. This observation highlights the need for objective risk stratification tools that can support referral decisions in patients undergoing cardiovascular intervention.

Among the total cohort, 1,227 patients had three consecutive positive FeOB results, of whom 258 (21.1 %) received endoscopy. The remaining patients did not undergo further endoscopy due to a history of hemorrhoids, consumption of animal blood before hospitalization, or personal preference. This rate is lower than that reported in US and Korean populations [7], [8], [9]. The relatively low compliance rate among FeOB-positive patients can be attributed to the invasive nature and high cost of endoscopy. In our study, the prevalence of GI MT was 39.9 % in the FeOB-positive group.

FeOB screening is a primary tool for detecting GI MT lesions. Due to the high prevalence of hemorrhoid in China, the FeOB may be positive among this population. Therefore, to eliminate the interference of hemorrhoids on the FeOB, more than once test is essential. Previous studies have reported that three-sample FeOB test can improve the specificity by 32 % compared to one-sample FeOB test [6]. Our study also revealed that the rate of FeOB turning negative at the second or third test was significantly lower in the GI MT group than in the non-GI MT group. In clinical settings, if patients have a history of hemorrhoids, it is essential to carefully exclude their impact on malignant tumor screening. The first and most economical approach is repeat FeOB testing. Sueta et al. [1] reported that among 232 FeOB positive patients before PCI, only 3 patients with early cancer lesions who underwent surgical treatment before PCI were detected. The study identified more benign lesions, such as adenomas and diverticula. There was no association between positive FeOB and lower intestinal malignant lesions. PCI was prioritized if patients had stable hemoglobin level over the preceding months or had undergone colonoscopy within the past year, among other factors.

Finally, in our study, we considered several potential risk factors, including age, gender, combination of medication usage, hemoglobin level and creatinine level，which could influence the accuracy of FeOB. Notably, we found that age and FeOB test for three times were significant factors associated with GI MT. As reported, unexplained anemia is regarded as a potential warning signal to screen GI MT [12], followed by endoscopy. Schneider et al. also found older age was strongly associated with colorectal cancer, with over 80 % of GI MT patients aged 60 years or older [13]. In multivariable regression model, after adjustment for age and FeOB test, hemoglobin was no longer independently associated with GI MT, since three consecutive positive FeOB tests were a much stronger predictor of GI MT. In our study, we established a nomogram，which provides a visual and quantitative tool for clinicians to estimate the probability of GI MT in individual patients. The DCA graph showed the net benefit of different prediction strategies (using age only, FeOB positive only, age + FeOB positive, “All” patients, or “None” patients as high-risk) across a range of high-risk thresholds. Net benefit declined as the high-risk threshold increased, but the combined model remained clinically useful when the threshold was <0.4. Linking to the nomogram and DCA graph, when the predicted probability of GI MT exceeds 0.2, further endoscopy is generally recommended. The ROC-derived age cutoff of 65.5 years provides a simple, memorable binary threshold for initial risk stratification in clinical settings. The nomogram, by utilizing age as a continuous variable, complements this by providing a more precise, individualized risk estimation, which is particularly valuable for risk assessment in patients whose age is near this cutoff value.

## Conclusions

In the real-world clinical settings, identifying patients with a high probability of GI MT is essential to avoid unnecessary DAPT discontinuation after PCI. Advanced age and persistent FeOB positivity are associated with increased risk of GI MT in patients undergoing evaluation prior to PCI. Risk estimation using a simple clinical model may support individualized decisions regarding endoscopic examination.

## Limitations

Firstly, the sample size of patients who underwent endoscopy was relatively small. Second, as noted, lifestyle factors (e.g., diet, alcohol consumption patterns beyond simple “yes/no” classification) and family history of GI malignancies, which are associated with GI MT risk, were not analyzed due to the lack of systematically recorded data in our retrospective design. Future prospective studies should incorporate these factors to develop more comprehensive prediction models. Third, not all patients received endoscope, there might be bias in the study. Fourth, as a retrospective study, the precise time interval between the positive FeOB test and the endoscopy was not recorded. Although institutional protocol generally recommends prompt endoscopic evaluation in this patient population, unmeasured variability in this interval could introduce potential bias. Additionally, our study did not perform subgroup analyses based on tumor location (e.g., gastric vs. colorectal). While this was beyond the scope of the present study, future research with larger cohorts could explore whether predictive factors differ by tumor site.

## Supplementary Material

Supplementary Material
