# Supplementary material for: Predictors of gastrointestinal malignant tumors in fecal occult blood positive individuals prior to percutaneous coronary intervention: a Nomogram-based approach
Source: Open Med (Wars). 2026 Jun 1;21(1):20261427. doi: 10.1515/med-2026-1427 (PMC13221252; doi:10.1515/med-2026-1427)
Supplement: Supplementary file 1 — Supplementary Material [file j_med-2026-1427_suppl_001.docx]

Supplementary Materials

**Table S1** Baseline characteristics between the 50 patients lost to follow-up and the 1,986 patients who remained in the study

|  | Lost to Follow-up  (n=50) | Included Cohort  (n=1986) | *P* value |
| --- | --- | --- | --- |
| Age(yrs) | 63.5(57.0~72.0) | 66.0(59.0~72.0) | *P*=0.20 |
| Male (n, %) | 38(76.0%) | 1545(77.8%) | *P*=0.73 |
| Hypertension (n, %) | 33 (66.0%) | 1314(66.2%) | *P*=1.00 |
| Diabetes mellitus (n, %) | 17(34.0%) | 582(29.3%) | *P*=0.53 |
| Smoking (n, %) | 23 (46.0%) | 883(44.5%) | *P*=0.89 |
| Alcohol (n, %) | 7(14.0%) | 233(11.7%) | *P*=0.66 |
| Prior PCI (n, %) | 19(38.0%) | 725(36.5%) | *P*=0.88 |
| Atrial fibrillation (n, %) | 3(6.0%) | 131(6.6%) | *P*=1.00 |
| Antiplatelet drug (n, %) |  |  |  |
| Aspirin | 39(78.0%) | 1305(65.7%) | *P*=0.07 |
| Cilostazol | 0(0%) | 35(1.8%) | *P*=1.00 |
| Clopidogrel | 15(30.0%) | 627(31.6%) | *P*=0.88 |
| Ticagrelor | 5(10.0%) | 286(14.4%) | *P*=0.54 |
| Oral anticoagulants (n, %) | 0(0%) | 51(2.6%) | *P*=0.63 |
| Proton pump inhibitors (n, %) | 26(52.0%) | 889(44.8%) | *P=*0.32 |
| Type of angina (n, %) |  |  | *P*=0.91 |
| SCAD | 33(66.0%) | 1348(67.9%) |  |
| UA | 6(12.0%) | 275(13.8%) |  |
| NSTEMI | 6(12.0%) | 206(10.4%) |  |
| STEMI | 5(10.0%) | 157(7.9%) |  |
| Hemoglobin(g/L) | 132.5(119.25~142.25) | 135.0(122.0~145.0) | *P=*0.23 |
| Platelet count(10^9^/L) | 184.5(157.5~230.75) | 201.0(167.75~243.0) | *P=*0.05 |
| Creatinine(µmol/L) | 81.0(71.75~93.25) | 80.0(70.0~93.0) | *P*=0.70 |
| Received PCI (n, %) | 36(72.0%) | 1566(78.9%) | *P*=0.29 |
| Gastric disease history (n, %) | 3(6.0%) | 307(15.5%) | *P*=0.06 |
| FeOB becomes negative  at second or third times (n, %) | 18(36.0%) | 759 (38.2%) | *P*=0.74 |

Values were presented in number (percentage) or normally distributed variables as mean±SEM, non-normally distributed as median (IQR)

Abbreviations: PCI, percutaneous coronary intervention; SCAD, stable coronary artery disease; UA, unstable angina; NSTEMI, Non-ST-Elevation Myocardial Infarction; STEMI, ST - Elevation Myocardial Infarction; FeOB, fecal occult blood;

**Table S2** Baseline characteristics between the patients who lost endoscopy and received endoscopy in the study

|  | Lost endoscopy  (n=1559) | Received endoscopy  (n=427) | *P* value |
| --- | --- | --- | --- |
| Age(yrs) | 66.0(58.0~71.0) | 67.0(61.0~73.0) | *P*＜0.001 |
| Male (n, %) | 1214(77.9%) | 331(77.5%) | *P*=0.90 |
| Hypertension (n, %) | 1037(66.5%) | 277(64.9%) | *P*=0.53 |
| Diabetes mellitus (n, %) | 456(29.2%) | 126(29.5%) | *P*=0.95 |
| Smoking (n, %) | 710 (45.5%) | 173(40.5%) | *P*=0.07 |
| Alcohol (n, %) | 180(11.5%) | 53(12.4%) | *P*=0.61 |
| Prior PCI (n, %) | 582(37.3%) | 143(33.5%) | *P*=0.16 |
| Atrial fibrillation (n, %) | 98(6.3%) | 33(7.7%) | *P*=0.32 |
| Hemoglobin(g/L) | 136.0(125.0~146.0) | 127.0(111.0~141.0) | *P*＜0.001 |
| Platelet count(10^9^/L) | 200.0(168.0~241.0) | 203.5(167.0~251.0) | *P=*0.16 |
| Creatinine(µmol/L) | 80.0(70.0~93.0) | 81.5(71.0~95.3) | *P*=0.10 |
| Gastric disease history (n, %) | 221(14.2%) | 86 (20.1%) | *P*＜0.01 |
| FeOB becomes negative  at second or third times (n, %) | 590(37.8%) | 169(39.6%) | *P*=0.48 |

Values were presented in number (percentage) or normally distributed variables as mean±SEM, non-normally distributed as median (IQR)；

Abbreviations: PCI, percutaneous coronary intervention; FeOB, fecal occult blood;

**Table S3** Univariable analysis showing associations between baseline clinical variables and GI MT

|  | Univariable model | |
| --- | --- | --- |
|  | Odds ratio (95% *CI*) | *P* value |
| Age | 1.04(1.02-1.07) | <0.01 |
| Gender | 1.18(0.71-1.96) | 0.52 |
| Hypertension | 0.92(0.60-1.43) | 0.92 |
| Diabetes mellitus | 0.97(0.62-1.53) | 0.97 |
| Smoking | 0.85(0.56-1.29) | 0.85 |
| Alcohol | 0.73(0.40-1.33) | 0.30 |
| Prior PCI | 1.40(0.89-2.20) | 0.14 |
| Atrial fibrillation | 0.97(0.45-2.11) | 0.97 |
| Antiplatelet drugs | 1.15(0.75-1.78) | 0.52 |
| Oral anticoagulants | 0.93(0.32-2.73) | 0.89 |
| Gastric disease history | 1.29(0.76-2.21) | 0.35 |
| Hemoglobin | 0.99(0.98-0.99) | <0.01 |
| Platelet | 1.00(0.99-1.01) | 0.12 |
| Creatinine | 1.00(0.99-1.01) | 0.88 |
| Proton pump inhibitors | 0.99(0.65-1.52) | 0.97 |
| Persistent FeOB_positivity | 4.02(2.44-6.61) | <0.01 |

Abbreviations: PCI, percutaneous coronary intervention; FeOB, fecal occult blood

a b


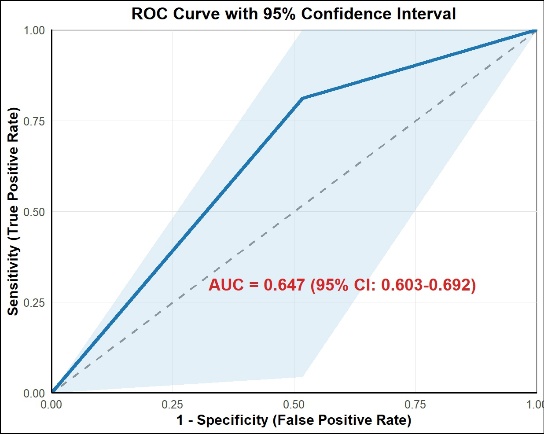

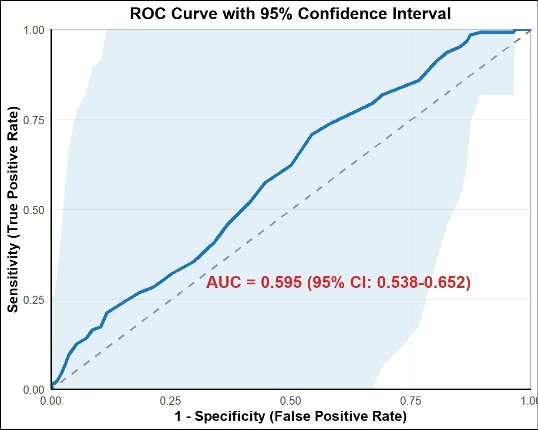


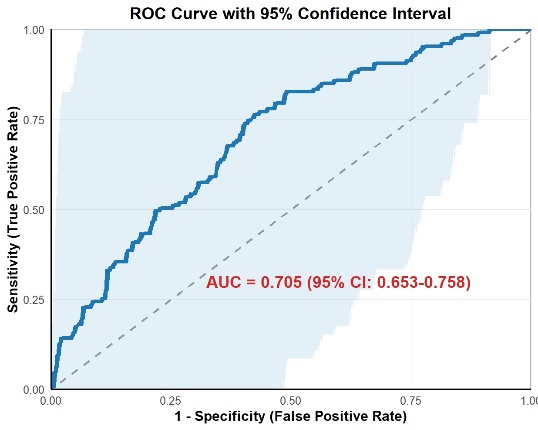

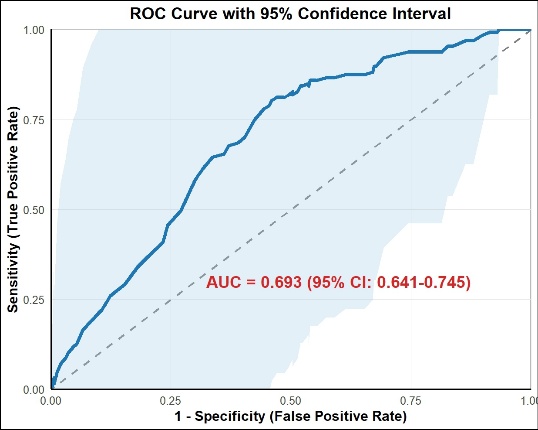
c d

**Figure S1** ROC curve for FeOB positive for three times(a), age(b), their combination(c) and the combination of the above two variables, along with Proton pump inhibitors usage and hemoglobin levels (d) in predicting GI MT.

Abbreviations: FeOB, fecal occult blood; GI MT, gastrointestinal malignant tumor.
